# Detection of ALK fusion transcripts in FFPE lung cancer samples by NanoString technology

**DOI:** 10.1186/s12890-017-0428-0

**Published:** 2017-05-26

**Authors:** Adriane F. Evangelista, Maicon F. Zanon, Adriana Cruvinel Carloni, Flávia E. de Paula, Mariana Andozia Morini, Maressa Ferreira-Neto, Iberê Cauduro Soares, Jose Elias Miziara, Pedro de Marchi, Cristovam Scapulatempo-Neto, Rui M. Reis

**Affiliations:** 1 Molecular Oncology Research Center, Barretos Cancer Hospital, Rua Antenor Duarte Villela, 1331, Barretos, CEP 14784-400 São Paulo Brazil; 2 Department of Pathology, Barretos Cancer Hospital, Rua Antenor Duarte Villela 1331, Barretos, CEP 14784-400 São Paulo Brazil; 3 Department of Thoracic Surgery, Barretos Cancer Hospital, Rua Antenor Duarte Villela 1331, Barretos, CEP 14784-400 São Paulo Brazil; 4 Department of Clinical Oncology, Barretos Cancer Hospital, Rua Antenor Duarte Villela 1331, Barretos, CEP 14784-400 Sao Paulo Brazil; 5 Life and Health Sciences Research Institute (ICVS), Health Sciences School, University of Minho, Braga, 4710-057 Portugal; 6 ICVS/3B’s-PT Government Associate Laboratory, Braga/Guimarães, 4710-057 Portugal

**Keywords:** *ALK*, *ALK* fusions, NanoString, FFPE

## Abstract

**Background:**

*ALK*-rearranged lung cancers exhibit specific pathologic and clinical features and are responsive to anti-*ALK* therapies. Therefore, the detection of *ALK*-rearrangement is fundamental for personalized lung cancer therapy. Recently, new molecular techniques, such as NanoString nCounter, have been developed to detect *ALK* fusions with more accuracy and sensitivity.

**Methods:**

In the present study, we intended to validate a NanoString nCounter *ALK*-fusion panel in routine biopsies of FFPE lung cancer patients. A total of 43 samples were analyzed, 13 ALK-positive and 30 ALK-negative, as previously detected by FISH and/or immunohistochemistry.

**Results:**

The NanoString panel detected the presence of the *EML4-ALK*, *KIF5B-ALK* and *TFG-ALK* fusion variants. We observed that all the 13 ALK-positive cases exhibited genetic aberrations by the NanoString methodology. Namely, six cases (46.15%) presented *EML-ALK* variant 1, two (15.38%) presented *EML-ALK* variant 2, two (15.38%) presented *EML-ALK* variant 3a, and three (23.07%) exhibited no variant but presented unbalanced expression between 5’/3’ exons, similar to other positive samples. Importantly, for all these analyses, the initial input of RNA was 100 ng, and some cases displayed poor RNA quality measurements.

**Conclusions:**

In this study, we reported the great utility of NanoString technology in the assessment of *ALK* fusions in routine lung biopsies of FFPE specimens.

**Electronic supplementary material:**

The online version of this article (doi:10.1186/s12890-017-0428-0) contains supplementary material, which is available to authorized users.

## Background

The *anaplastic lymphoma kinase* (*ALK*) gene is located in the 2p23 chromosome region and codifies a tyrosine kinase receptor of the insulin receptor family [1]. Genetic aberrations in the *ALK* gene were first described in anaplastic large cell lymphoma by Morris and colleagues [1] as part of an oncogenic fusion protein resulting from the translocation between chromosomes 2 and 5 (t[2;5] [p23;q35]) (*NPM-ALK - Nucleophosmin- anaplastic lymphoma kinase*). In 2007, Soda and colleagues concluded that the *ALK* gene fusion was also an oncogenic driver of non-small cell lung cancer (NSCLCs) [2]. In these tumors, the most common fusion partner, the *EML4* (*Echinoderm microtubule-associated protein-like 4*) gene, results from a small inversion within the short arm of chromosome 2, and it is found in approximately 3–7% of NSCLCs [2, 3]. Other partners encoding *KIF5B-ALK* (<1%), *TFG-ALK* (2%), *KLC1-ALK* (<5%), *PTPN3-ALK* (<1%) and in rare events and isolated cases the aberrant proteins *HIP1-ALK*, *TPR-ALK*, *STRN-ALK*, and *A2M-ALK* have also been described in cancers [4–6]. The aberrant *ALK* fusions promote dimerization domains that are ligand independent, with consequent constitutive kinase activity and malignant transformation [5].

*ALK*-rearranged lung cancers exhibit specific clinical and pathologic features [7]. They are associated with younger patients (<50 years), non-smokers, prevalent mucinous histology, signet ring morphology in some cases, and *EGFR* and *KRAS* wild-type cases [3, 8, 9]. Importantly, *ALK* rearrangement predicts the clinical response to Crizotinib (Xalkori®, Pfizer), an oral MET/ALK inhibitor, ATP-competitive inhibitors of the ALK tyrosine kinase, Ceritinib (Zykadia™, Novartis) and Alectinib (Alecensa®, Roche), second-generation *ALK* inhibitors, and lorlatinib (PF-06463922) [10, 11].

Consequently, accurate molecular methodologies that detect and quantify *ALK* fusions and their variants have been developed for therapeutic selection. Recently, an *ALK* fusion panel was designed and tested using NanoString technology, which is considered a more sensitive method since it performs direct molecule counting, avoiding the bias associated with amplification [12–14]. The *ALK* panel was created using two strategies. The first group of eight probes over several exons was designed to detect an unbalanced expression. The second set of probes was designed in the breakpoint of the known variants of *EML4-ALK*, *KIF5B-ALK* and *TFG-ALK*. For this strategy, a pair of adjacent probes (35–50 bp each) were used, the first (biotin-capture probe) complementary to the partner gene, and the other (barcoded reporter probe) to the *ALK* gene at exon 20 [13].

In the present study, we reported the feasibility of the NanoString *ALK* fusion panel to detect the *ALK* fusion transcripts in formalin-fixed paraffin-embedded (FFPE) samples of lung adenocarcinoma in a Brazilian population. We also evaluated whether a lower quantity (up to 100 ng) of RNA could be used in a routine diagnostic setting of tumor biopsies.

## Methods

### Material

In the present study, we performed a retrospective evaluation of a convenience series of 43 lung carcinoma samples from the Pathology Department of Barretos Cancer Hospital (Brazil) between 2012 and 2015 (Additional file 1: Table S1). These cases were selected based on their previous evaluation for *ALK* rearrangement by immunohistochemistry and/or fluorescence in situ hybridization (FISH) in our Department of Pathology. The cases were also assessed for *EGFR* and *KRAS* mutation status.

As controls, we used the lung adenocarcinoma cell lines H2228 (ALK-positive) and CALU3 (ALK-negative) (ATCC, Manassas, VA). Cell monolayers were grown in Dulbecco’s modified Eagle’s medium (DMEM) (Gibco) supplemented with 10% fetal bovine serum (Gibco) and 1% penicillin-streptomycin (Gibco). The cells were incubated in a 5% CO_2_ environment at 37 °C.

This study was approved by the Barretos Cancer Hospital Ethical Review Committee ((#630/2012). Due the retrospective nature of the study, the Local Ethical Review Committee waived the need for patients written informed consent.

### DNA isolation

Serial 5-μm unstained sections of formalin-fixed paraffin-embedded blocks were cut, and one adjacent hematoxylin and eosin-stained (H&E) section was taken for pathologist identification and selection of the tumor tissue. DNA was macrodissected from 1 unstained section from each specimen as previously described [15, 16]. Briefly, tissues were deparaffinized at 80 °C and serially washed with xylene and ethanol (100, 70 and 50%). Selected areas of the tumor or precursor lesions were macrodissected using a sterile needle (18G × 1 ^1^/^2^) (BD, Curitiba, Brazil) and carefully collected into a microtube. DNA was extracted using a QIAamp DNA Micro Kit (Qiagen, Hilden, Germany) following the manufacturer’s instructions. DNA quantity and quality was evaluated by Nanodrop 2000 (Thermo Scientific, Wilmington, USA). DNA samples were diluted to a final concentration of 50 ng/μl and stored at −20 °C for further molecular analysis.

### Mutational analysis of *EGFR* and *KRAS*

The hotspot regions of the oncogenes *EGFR* (exons 18, 19, 20 and 21) and *KRAS* (codons 12/13) were analyzed by polymerase chain reaction (PCR), followed by direct sequencing, as previously described [17]. Briefly, PCR was performed in a final volume of 15 μl, with 50 ng of DNA and 10 μM of forward and reverse primers, using 7.5 μl of the HotStar master mix (Qiagen, Hilden, Germany) according to the protocol proposed by the manufacturer, with the following cycling parameters: 96 °C for 15 min, followed by 40 cycles of 96 °C for 45 s, 58 °C for 45 s (*EGFR*) or 56.5 °C for 45 s (*KRAS*), 72 °C for 45 s and 72 °C for 10 min in a thermocycler (Veriti, Applied Biosystems, Carlsbad, USA). Primer sequences were previously described [15]. The PCR products were evaluated by electrophoresis in agarose gel and further purified using ExoSAP-it (Affymetrix), followed by cycle sequencing carried out using a BigDye Terminator v3.1 Cycle Sequencing kit (Applied Biosystems, Foster City, CA) with an initial denaturation at 97 °C for 3 min, followed by 28 cycles of 96 °C for 10 s, 50 °C for 5 s, and 60 °C for 4 min. Sequencing products were purified using BigDye Xterminator (Applied Biosystems) and analyzed on a 3500 DNA Analyzer with a ABI capillary electrophoresis system (Applied Biosystems). Sequences were analyzed using the SeqScape software package (Applied Biosystems).

### Fluorescence *in situ* hybridization (FISH) Assay

The presence of *ALK* gene rearrangement was determined using fluorescence *in situ* hybridization (FISH) assay using the commercially available ALK probe (Vysis LSI ALK Dual Color, Break Apart Rearrangement Probe; Abbott Molecular, Abbott Park, IL) as described elsewhere [18]. The lung adenocarcinoma cell lines H2228 (ALK-positive) and CALU3 (ALK-negative) were used as the positive and negative control, respectively. Paraffin-embedded sections (5 μm thick) were initially incubated at 60 °C, deparaffinized with xylene and dehydrated in 100% ethanol. Tissue sections were then transferred to a 0.2 N HCL solution and incubated at 80 °C with 10 mM citrate at pH 6.4 and 1 mM EDTA at pH 8.0. Slides were enzymatically treated with pepsin in 0.01 N HCL and washed once with water. Five microliters of the ALK probe was applied to the tumor tissue, and a subsequent denaturation step was performed at 83 °C for 10 min. Hybridization was carried out for 22 h in a humidified chamber at 37 °C. Tissue sections were washed in 0.3% Igepal/2x SSC at 63 °C for 4 min, and then washed with 2x SSC at room temperature. Nuclei were counterstained with DAPI (4’,6-diamidino-2-phenylindole) and analyzed using an Eclipse 50i microscope with fluorescence (Nikon Instruments). From each sample, 100 tumor nuclei per slide were analyzed, and the standard score of > = 15% was used to determine the presence of ALK rearrangement, as previously reported [18, 19]. For the analysis, the software FISHView 6.0 (Applied Spectral Imaging) was used.

### Immunohistochemistry (IHC)

The presence of ALK overexpression was assessed by immunohistochemistry (IHC) staining using 4-μm-thick sections. Ganglion cells present in sections of the appendix were used as positive controls, and in negative controls, the primary antibody was omitted. Immunohistochemical reactions were performed at Ventana Benchmark XT using the Ventana ALK (D5F3) CDx assay (Ventana, Tucson, AZ, clone 790–4796) according to the manufacturer. In brief, slides of the NSCLC tumor were subjected to deparaffinization using EZ Prep (Ventana, Tucson, AZ) and antigen retrieval was performed using Cell Conditioning 1 (Ventana, Tucson, AZ). Tissue sections were then incubated with anti-ALK antibody (clone D5F3, Ventana, Tucson, AZ) for 20 min. The OptiView DAB IHC Detection Kit (Ventana, Tucson, AZ) and OptiView Amplification Kit (Ventana, Tucson, AZ) were used according to the manufacturer’s recommendations for the visualization of the bound primary antibody. The ALK stain was considered positive if at least one cell presented strong dark brown cytoplasmic staining as stated in the kit’s manual as previously described [20].

### RNA isolation

Tumor cell content (>60%) was assessed based on H&E-stained slides, and RNA was isolated from two to four sections (10 μm thick) using a RecoverALL total Nucleic Acid Isolation kit (Life Technologies) according to the manufacturer’s instructions. Briefly, the process is divided into four steps: (i) an initial preparation that includes slide scraping, deparaffinization with xylene and 100% ethanol dehydration; (ii) protease digestion; (iii) nucleic acid isolation using the filter cartridge, followed by washing; and (iv) DNase digestion with additional washes and elution. RNA concentration was assessed using both the Nanodrop 1000 spectrophotometer (NanoDrop Products, Thermo Scientific, Wilmington, DE) and Qubit (Life Technologies).

### NanoString nCounter Assay

The custom ALK panel was carried out using the NanoString nCounter Elements™ protocol per the manufacturer’s instructions. All procedures regarding sample preparation, hybridization, detection and scanning were performed as recommended by NanoString Technologies (NanoString, Seattle, WA). The custom probes (A and B) were designed by IDT (IDT Technologies, Coralville, USA) and contained 35–50 bp each, as previously described by Lira and co-authors [13]. Probes were diluted to a final concentration of 0.6 nM (probe A) and 3.0 nM (probe B) to create the 30X working probe pools. The total amount of up to 100 ng RNA was used. RNA was hybridized with probe pools, hybridization buffer and TagSet reagents in a total volume of 30 μl and incubated at 67 °C for 20 h. Samples were then loaded to the automated nCounter Sample Prep Station (NanoString Technologies, Seattle, WA), which performed the purification steps and cartridge preparation. Finally, the cartridges containing immobilized and aligned reporter complexes were transferred to a nCounter Digital Analyzer (NanoString Technologies), and expression data were subsequently generated using the high-resolution setting, which takes 600 images per sample.

### Data analysis

The reporter counts were collected using NanoString’s nSolver analysis software v2.5 and normalized as previously described by Lira and co-authors [13]. Briefly, raw probe counts were normalized by positive reaction controls to a panel of three housekeeping genes (*GUSB1*, *OAZ1* and *POLR2A*). Additionally, a detection 3’/5’ score was defined by the ratio between geometric mean of 3’-probes and average of 5’-probes, with a 2.0 threshold for positivity, as reported by Lira and co-workers [13]. The statistical-mathematical R software v.3.2.3 (https://www.r-project.org) was used for this analysis and graphical construction.

## Results

**Fig. 1:**
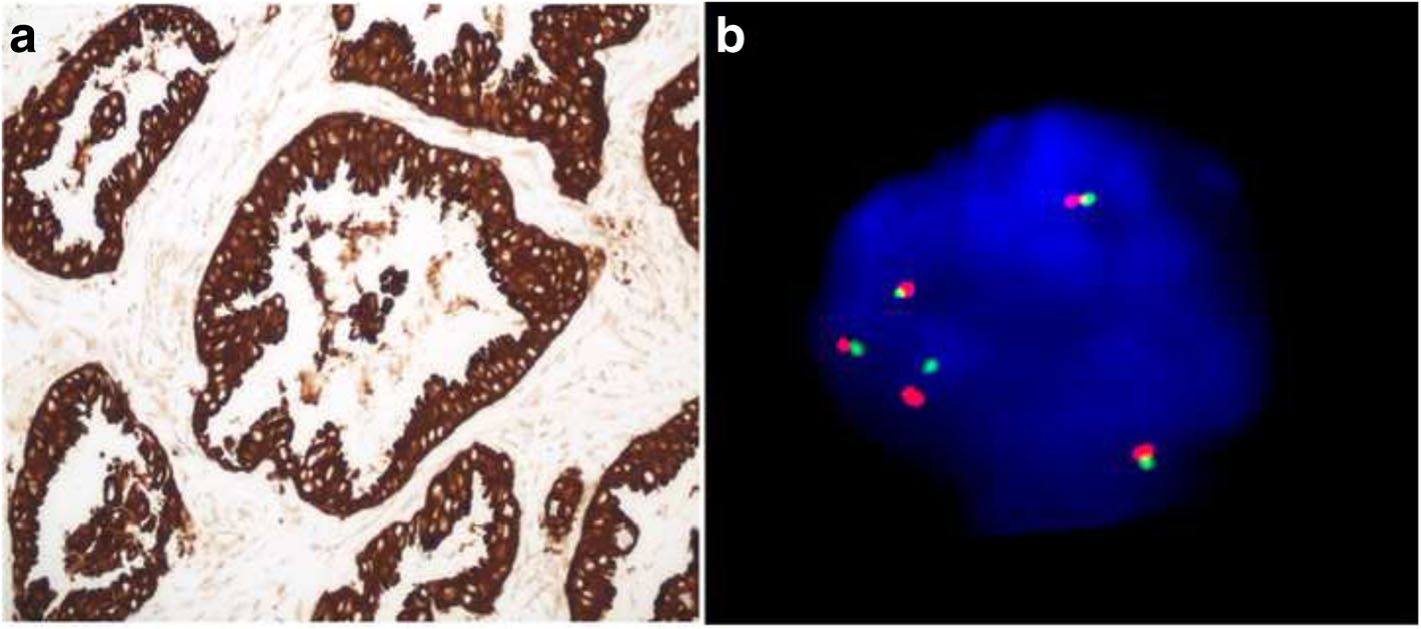
ALK positivity represented by IHC (**a**) and the interphase nucleus with the presence of ALK rearrangement by FISH (score >15%) (**b**)

All 43 cases analyzed were previously evaluated by immunohistochemistry (Fig. 1a), which showed the presence of ALK positivity in 13/43 (30.2%) of cases. In a subset of cases (*n* = 24), we also performed FISH assays (Fig. 1b). In these 24 cases, we found 100% concordance between the FISH and immunohistochemistry methodologies.

**Fig. 2:**
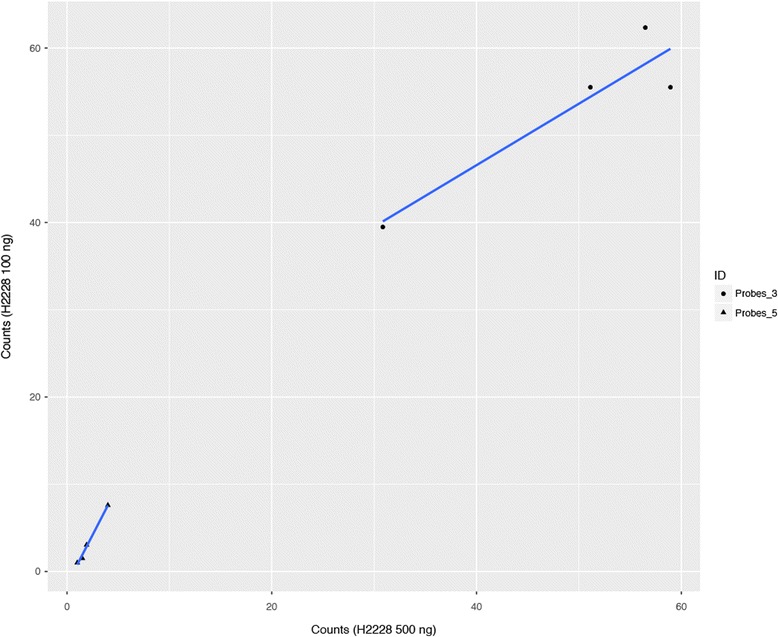
NanoString correlation between the initial input of 100 or 500 ng in the H2228 cell line

Concerning the NanoString assay, we first performed a pilot analysis to determine whether a distinct initial RNA quantity would yield equivalent results. Using the H2228 (*ALK*-positive) cell line, we compared the use of 500 ng of RNA as reported by Lira and co-authors [13] and 100 ng of RNA as recently suggested by NanoString. We reported Pearson correlations of 0.99 for 5’-probes and 0.93 for 3’-probes, with counts of *EML-ALK V3a* of 54.68 (100 ng) and 49.40 (500 ng), respectively (Fig. 2).

**Fig. 3:**
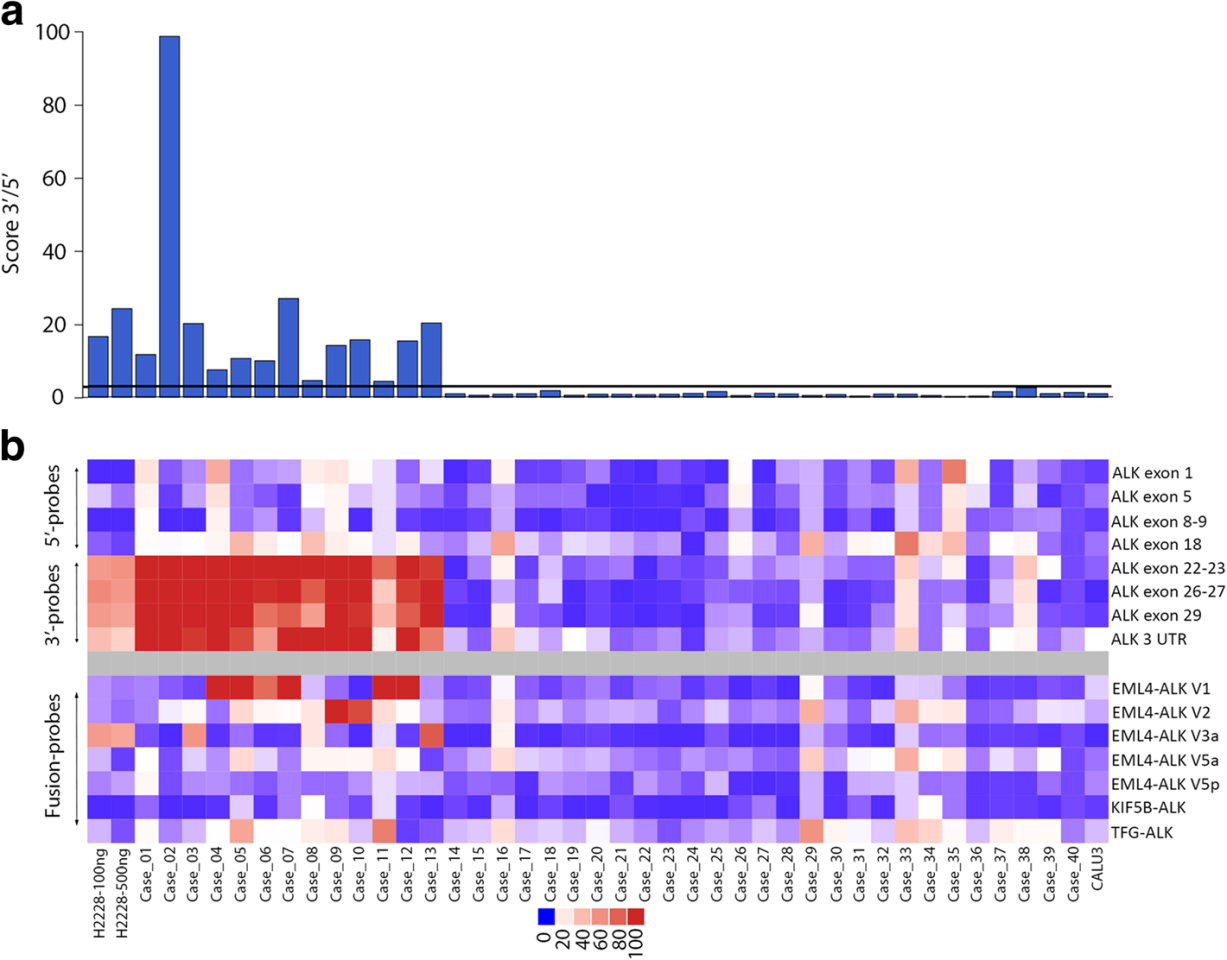
Barplot representing the ALK 3’/5’ score of each sample with the indication of the threshold line of positivity (**a**); Heatmap showing the expression of 5’-ALK probes (exon 1 to exon 18) and 5’-*ALK* probes (exon 22 to 3’-UTR) and ALK variants (*EML-ALK*, *KIF5B-ALK* and *TGF-ALK*) (**b**)

We further analyzed 43 cases, and of those, three were excluded due to signal detection flags by NanoString nSolver software. These three cases were from biopsies and two of them yielded a total RNA quantity below 100 ng (Additional file 1: Table S1). The 3’/5’ score and heatmap of normalized expression values of validated cases (*n* = 40) are shown in Fig. 3a and b, respectively. The concordance between the NanoString, immunohistochemistry and FISH methodologies was 100%.

Among the 13 *ALK*-positive cases, the following variants were identified: six (46.15%) exhibited *EML-ALK 1*, two (15.38%) exhibited *EML-ALK 2*, two (15.38%) exhibited *EML ALK-V3a*, and three (23.07%) exhibited no variant. However, in these cases, the increase in counts in 3’-probes was evident, with average 3’/5’ scores of approximately 20 and normalized expression values of approximately 100 (case 1 to 13) (Fig. 3a and b). The variant *EML-ALK-V3a* presented lower expression than the others (case_3 and case_13), accounting for 57.61 and 82.67 of normalized NanoString counts, respectively. These normalized values were similar to those observed for the H2228 cell line, which also exhibited this variant (Fig. 3b). No differences in *KIF5B-ALK* variants were observed in the cases analyzed. *TFG-ALK* presented normalized values of approximately 40 in two cases (Fig. 3b). However, this was not considered relevant since the same patients presented higher counts of *EML-ALK 1*. Moreover, similar normalized values of *TFG-ALK* were also observed in *ALK*-negative patients (Fig. 3b).

## Discussion

Aberrant *ALK* fusions were recently identified in a subset of lung cancer patients, resulting in the constitutive activation of MEK/ERK and PI3K pathways, with consequent up-regulation of cell survival and proliferation mechanisms [9, 21]. Importantly, its detection had a paramount clinical impact, as it was a predictive biomarker of the therapeutic response [9, 21]. Recently, Lira and co-authors [13] design a panel to detect *ALK* fusion rearrangements by the NanoString assay. The authors analyzed 34 *ALK*-positive and 33 *ALK*-negative FFPE non-small cell lung cancer samples, obtaining 93% concordance with FISH and 98% concordance with immunohistochemistry [13]. For these analyses, the authors used 500 ng of input RNA, which is an important value. In the diagnostic procedures for lung cancer pathology, the availability of sufficient quantities of biopsy tissue ultimately limit the application of these methodologies.

In the present study, we sought to implement the NanoString panel of *ALK* fusion detection for lung cancer patients and to optimize its applicability in biopsies using up to 100 ng of RNA. These small amounts are in line with the recent protocols of NanoString for Elements. We analyzed the biopsy specimens of 43 patients diagnosed with lung cancer, mainly non-small cell lung cancer. Following RNA isolation, we observed the heterogeneity of RNA quality and quantity, representing the reality of a routine setting. Despite the low quantities of RNA used and their variable quality, only three cases were flagged (3 *ALK*-negative), representing just ~7% failure. The improvement of the panel, including the detection of the clearly positive samples (according to 5’/3’ unbalanced probes) but detecting no variant, can also be of potential use. Our NanoString results were further compared with FISH and immunohistochemistry analysis and showed a full concordance of methodologies. Of the 40 cases with reliable results, 13 exhibited *ALK* gene fusions. *EML-ALK 1* was the most common, being present in approximately half of them. These results are in agreement with previous studies [4, 5].

It has been reported that the different variants can exhibit distinct sensitivity to Crizotinib [22, 23]. Among the methodologies currently available for *ALK* fusion detection, only NanoString and next-generation sequencing (NGS) can perform such differentiation (Table 1). Recently, Rogers and co-authors compared the technics of FISH, NanoString, Agena LungFusion panel and ThermoFisher NGS [24]. The authors found a great concordance between all techniques, and reported that NanoString requires more RNA input, yet, is less prone to false-positives [24]. Moreover, Ali and co-authors [25] suggested that NGS could be especially useful for detecting ALK rearrangements, including those with other partners. One of the major advantages of NGS is the potential for multiplex testing, but it has some disadvantages, such as the higher cost, and it requires more effort for analysis [26] (Table 1).

**Table 1:** Comparison of FISH, IHC, qRT-PCR, NGS and NanoString regarding costs, procedural difficulty and resolution

		FISH	IHC	qRT-PCR	NGS	NanoString
Costs	Overal reagents costs	*/**	***	**/***	*	**/***
	Hardware & software costs	**	***	**	*	*
Procedural difficulty	RNA input required	NA	NA	*	***	**
	Poor quality samples	*/**	**/***	*	*/**	**/***
	Hands on time	*/**	*/**	**	*/**	***
	Run time and results analysis	**	**	**	*	**/***
	Interpretation of results	*/**	*/**	**	***	**
	Throughput	*	*/**	**	***	**/***
Resolution	Accuracy at low concentrations	NA	NA	**	*/**	***
	Quantitative precision	*/**	*/**	**	***	***
	Variants detection	*	*	**	***	**/***

*-worse option; **-moderate option; *** - best option: *NA* Not applicable

Furthermore, in Table 1, we address all current ALK rearrangement methodologies and explore their advantages and disadvantages. As shown in Table 1, NanoString represents an interesting option considering its hands-on time, quantitative precision, robustness in samples with extremely poor quality (since it is unbiased toward amplification and sequence errors) and ease of analysis. This technique allows for the detection of a high number of molecules similarly to microarrays, yet with a sensitivity for qPCR [14]. Moreover, NanoString is very flexible for the construction of custom panels, and it yields highly confident, accurate and reproductive results in low quality and quantity RNA from FFPE tissues [14]. However, it is important to consider that this new technology has some limitations, i) the high cost for hardware and software, mainly for the process of acquisition and maintenance of the devices, which has been limited in Latin America (Table 1); and ii) considering the fusion assay, the detection is mainly detected by a previously defined 3’/5’ score, which is not totally defined and it is not possible to detect new fusion partners. In this regard, further studies are necessary to expand the panel intending to include new partners and to develop a defined analysis pipeline for fusion detection, with specific thresholds for fusion probes to avoid bias of interpretation.

Finally, in the future, with the novel standardizations and improvements in chemistry to improve the sensitivity at low input when compared with other technics [24] (Table 1), NanoString methodology can be used to address special issues, such as the detection of ALK rearrangements in CTCs [27].

## Conclusions

In this study, we report the utility of NanoString technology in the assessment of *ALK* fusions in FFPE specimens from routine lung biopsies.

## Additional file

Additional file 1: Table S1. Association of ALK fusion and the main clinico-pathological and molecular features. Summary of the major clinico-pathological characteristics of patients and RNA quality information and ALK, EGFR and KRAS status. (XLSX 14 kb)

## Abbreviations

*A2M-ALK*: *alpha-2-macroglobulin* and *anaplastic lymphoma kinase*
*ALK*: *Anaplastic lymphoma kinase*
DAPI: 4’,6-Diamidino-2-phenylindole
DMEM: Dulbecco’s modified Eagle’s medium
*EML4-ALK*: *Echinoderm microtubule-associated protein-like 4* and *anaplastic lymphoma kinase*
FFPE: Formalin-fixed paraffin-embedded
FISH: Fluorescence in situ hybridization
H&E: Hematoxylin and eosin-stained
*HIP1-ALK*: *Huntingtin interacting protein 1* and *anaplastic lymphoma kinase*
IHC: Immunohistochemistry (IHC)
*KIF5B-ALK*: *Kinesin family member 5B* and *anaplastic lymphoma kinase*
*KLC1-ALK*: *Kinesin light chain 1* and *anaplastic lymphoma kinase*
*NPM-ALK*: *Nucleophosmin-anaplastic lymphoma kinase*
NSCLC: Non-small cell lung cancer
*PTPN3-ALK*: *Protein tyrosine phosphatase, non-receptor type 3* and *anaplastic lymphoma kinase*
*STRN-ALK*: *Striatin* and *anaplastic lymphoma kinase*
*TFG-ALK*: *TRK-fused gene* and *anaplastic lymphoma kinase*
*TPR-ALK*: *Translocated promoter region, nuclear basket protein* and *anaplastic lymphoma kinase*

## References

[1] Morris SW, Kirstein MN, Valentine MB, Dittmer KG, Shapiro DN, Saltman DL (1994). Fusion of a kinase gene, ALK, to a nucleolar protein gene, NPM, in non-Hodgkin’s lymphoma. Science.

[2] Soda M, Choi YL, Enomoto M, Takada S, Yamashita Y, Ishikawa S (2007). Identification of the transforming EML4-ALK fusion gene in non-small-cell lung cancer. Nature.

[3] Wong DW-S, Leung EL-H, So KK-T, Tam IY-S, Sihoe AD-L, Cheng L-C (2009). The EML4-ALK fusion gene is involved in various histologic types of lung cancers from nonsmokers with wild-type EGFR and KRAS. Cancer.

[4] Takeuchi K, Choi YL, Togashi Y, Soda M, Hatano S, Inamura K (2009). KIF5B-ALK, a novel fusion oncokinase identified by an immunohistochemistry-based diagnostic system for ALK-positive lung cancer. Clin Cancer Res.

[5] Rikova K, Guo A, Zeng Q, Possemato A, Yu J, Haack H (2007). Global Survey of Phosphotyrosine Signaling Identifies Oncogenic Kinases in Lung Cancer. Cell.

[6] Roskoski R (2013). Anaplastic lymphoma kinase (ALK): structure, oncogenic activation, and pharmacological inhibition. Pharmacol Res.

[7] Mossé YP, Wood A, Maris JM (2009). Inhibition of ALK signaling for cancer therapy. Clin Cancer Res.

[8] Gaughan EM, Costa DB (2011). Genotype-driven therapies for non-small cell lung cancer: focus on EGFR, KRAS and ALK gene abnormalities. Ther Adv Med Oncol.

[9] Shaw AT, Yeap BY, Mino-Kenudson M, Digumarthy SR, Costa DB, Heist RS (2009). Clinical features and outcome of patients with non-small-cell lung cancer who harbor EML4-ALK. J Clin Oncol.

[10] McKeage K (2014). Alectinib: A Review of Its Use in Advanced ALK-Rearranged Non-Small Cell Lung Cancer. Drugs.

[11] Wu J, Savooji J, Liu D (2016). Second- and third-generation ALK inhibitors for non-small cell lung cancer. J Hematol Oncol.

[12] Jung S-H, Sohn I (2014). Statistical Issues in the Design and Analysis of nCounter Projects. Cancer Inform.

[13] Lira ME, Kim TM, Huang D, Deng S, Koh Y, Jang B (2013). Multiplexed gene expression and fusion transcript analysis to detect ALK fusions in lung cancer. J Mol Diagn.

[14] Kulkarni MM (2011). Digital multiplexed gene expression analysis using the NanoString nCounter system. Curr Protoc Mol Biol.

[15] Yamane LS, Scapulatempo-Neto C, Alvarenga L, Oliveira CZ, Berardinelli GN, Almodova E (2014). KRAS and BRAF mutations and MSI status in precursor lesions of colorectal cancer detected by colonoscopy. Oncol Rep.

[16] Becker AP, Scapulatempo-Neto C, Carloni AC, Paulino A, Sheren J, Aisner DL (2015). KIAA1549: BRAF Gene Fusion and FGFR1 Hotspot Mutations Are Prognostic Factors in Pilocytic Astrocytomas. J Neuropathol Exp Neurol.

[17] Reis-Filho JS, Pinheiro C, Lambros MBK, Milanezi F, Carvalho S, Savage K (2006). EGFR amplification and lack of activating mutations in metaplastic breast carcinomas. J Pathol.

[18] Camidge DR, Kono SA, Flacco A, Tan A-C, Doebele RC, Zhou Q (2010). Optimizing the detection of lung cancer patients harboring anaplastic lymphoma kinase (ALK) gene rearrangements potentially suitable for ALK inhibitor treatment. Clin Cancer Res.

[19] Kwak EL, Bang Y-J, Camidge DR, Shaw AT, Solomon B, Maki RG (2010). Anaplastic lymphoma kinase inhibition in non-small-cell lung cancer. N Engl J Med.

[20] Wynes MW, Sholl LM, Dietel M, Schuuring E, Tsao MS, Yatabe Y (2014). An international interpretation study using the ALK IHC antibody D5F3 and a sensitive detection kit demonstrates high concordance between ALK IHC and ALK FISH and between evaluators. J Thorac Oncol.

[21] Wilson FH, Johannessen CM, Piccioni F, Tamayo P, Kim JW, Van Allen EM (2015). A functional landscape of resistance to ALK inhibition in lung cancer. Cancer Cell.

[22] Heuckmann JM, Balke-Want H, Malchers F, Peifer M, Sos ML, Koker M (2012). Differential protein stability and ALK inhibitor sensitivity of EML4-ALK fusion variants. Clin Cancer Res.

[23] Crystal AS, Shaw AT (2012). Variants on a theme: a biomarker of crizotinib response in ALK-positive non-small cell lung cancer?. Clin Cancer Res.

[24] Rogers T-M, Arnau GM, Ryland GL, Huang S, Lira ME, Emmanuel Y (2017). Multiplexed transcriptome analysis to detect ALK, ROS1 and RET rearrangements in lung cancer. Sci Rep.

[25] Ali SM, Hensing T, Schrock AB, Allen J, Sanford E, Gowen K (2016). Comprehensive Genomic Profiling Identifies a Subset of Crizotinib-Responsive ALK-Rearranged Non-Small Cell Lung Cancer Not Detected by Fluorescence In Situ Hybridization. Oncologist.

[26] Dagogo-Jack I, Shaw AT (2016). Screening for ALK Rearrangements in Lung Cancer: Time for a New Generation of Diagnostics?. Oncologist.

[27] Cesano A (2015). nCounter(®) PanCancer Immune Profiling Panel (NanoString Technologies, Inc., Seattle, WA). J Immunother Cancer.

